# Towards neuroadaptive chatbots: a feasibility study

**DOI:** 10.3389/fnrgo.2025.1589734

**Published:** 2025-10-15

**Authors:** Diana E. Gherman, Thorsten O. Zander

**Affiliations:** ^1^Chair of Neuroadaptive Human-Computer Interaction, Brandenburg University of Technology Cottbus-Senftenberg, Cottbus, Germany; ^2^Zander Laboratories GmbH, Cottbus, Germany

**Keywords:** passive brain-computer interfaces, pBCI, LLM, error-processing, moral judgment, AI alignment

## Abstract

**Introduction:**

Large-language models (LLMs) are transforming most industries today and are set to become a cornerstone of the human digital experience. While integrating explicit human feedback into the training and development of LLM-based chatbots has been integral to the progress we see nowadays, more work is needed to understand how to best align them with human values. Implicit human feedback enabled by passive brain-computer interfaces (pBCIs) could potentially help unlock the hidden nuance of users' cognitive and affective states during interaction with chatbots. This study proposes an investigation on the feasibility of using pBCIs to decode mental states in reaction to text stimuli, to lay the groundwork for neuroadaptive chatbots.

**Methods:**

Two paradigms were created to elicit moral judgment and error-processing with text stimuli. Electroencephalography (EEG) data was recorded with 64 gel electrodes while participants completed reading tasks. Mental state classifiers were obtained in an offline manner with a windowed-means approach and linear discriminant analysis (LDA) for full-component and brain-component data. The corresponding event-related potentials (ERPs) were visually inspected.

**Results:**

Moral salience was successfully decoded at a single-trial level, with an average calibration accuracy of 78% on the basis of a data window of 600 ms. Subsequent classifiers were not able to distinguish moral judgment congruence (i.e., moral agreement) and incongruence (i.e., moral disagreement). Error processing in reaction to factual inaccuracy was decoded with an average calibration accuracy of 66%. The identified ERPs for the investigated mental states partly aligned with other findings.

**Discussion:**

With this study, we demonstrate the feasibility of using pBCIs to distinguish mental states from readers' brain data at a single-trial level. More work is needed to transition from offline to online investigations and to understand if reliable pBCI classifiers can also be obtained in less controlled language tasks and more realistic chatbot interactions. Our work marks preliminary steps for understanding and making use of neural-based implicit human feedback for LLM alignment.

## 1 Introduction

AI-powered chatbots are becoming a ubiquitous part of the modern human experience. Since the tech company OpenAI deployed the ChatGPT^1^ model in November 2022, numerous ever-increasingly powerful large language models (LLMs) have been created, with new releases and improvements being announced weekly. LLMs are now widely used by knowledge workers for summarization, brainstorming, information organizing and searching, coding, and more. To this end, recent surveys have shown a stark improvement in productivity and quality of work for people who get assistance from chatbots (Noy and Zhang, 2023). Outside of working hours, people realized LLMs can serve not only as tutors and assistants but also as counselors who can help with solving conflicts, offer relationship advice (Vowels, 2024), or help understand oneself better (Giubilini et al., 2024). Several companies aimed to seize the rising tide of opportunity and developed LLM-based products meant to offer friendly and romantic companionship (Babu and Prasad, 2024), or therapeutic personas (Haque and Rubya, 2023). Despite the ease of access to such services, a recent review into the effectiveness of LLM-based mental health applications concludes that “current risks associated with clinical use might surpass their benefits” due to their tendency to offer inconsistent advice and hallucinate, which might result in more harm and confusion for the end user (Guo et al., 2024). This concern is not only limited to mental health services, but applies broadly to many areas that require a deep understanding of human emotions and perception, empathy, and contextual judgment (Huang et al., 2023). For example, an intricate cognitive and emotional understanding of the human mind is needed to excel at tasks related to education, ethics, or diplomacy. Such qualities remain difficult for LLMs to fully replicate, rendering a subtle gap between man and machine that becomes increasingly apparent in situations requiring moral reasoning and emotional nuance. While recent technical developments in the Natural Language Processing (NLP) domain have led to better LLM soft skills, there is still room for improvement (Sorin et al., 2024).

The call for human-AI alignment started early in the development of AI but has remained a fringe topic until recently. The alignment problem refers to the challenge of keeping AI systems consistent with human values, intentions, and preferences as they become more intelligent and complex. It also involves that no unintended and harmful consequences arise as AI systems scale (Russell, 2019). With the advent of powerful LLMs and empirical demonstrations of misaligned AI behavior that was once just theory and speculation (Meinke et al., 2024; Pan et al., 2024), this call has amplified. The daily mainstream tech news titles now constitute a mix of *Terminator-*type doomsday predictions (The Independent, 2023) and the latest winner of the world AI race. The concerned voices are especially louder nowadays due to the emergence of agentic AI systems, which are potentially soon to take over the oracle-type of AI tools used today (Chan et al., 2023). Consequently, companies and governments are actively working on integrating into digital infrastructures AI systems that are designed to act and make decisions autonomously, without human input. It therefore becomes imperative to address misalignment concerns and explore new ways to enhance machines' understanding of human goals and values. So far, utilizing human feedback within the training of AI models has been the main approach for LLM alignment (Ziegler et al., 2019), as well as a crucial factor behind ChatGPT's overwhelming success. Reinforcement Learning with Human Feedback (RLHF) has been employed to effectively replace the standard reward signals in reinforcement learning with explicit feedback meant to symbolize the quality of an LLM's output in terms of criteria such as relevance, factual accuracy, coherence, and even adherence to ethical standards (Li et al., 2024). As a cognition-driven approach, RLHF tries to decipher the underlying user intent that guides the preferred responses in language models and then steer the model to generate outputs that align with that intent (Chaudhari et al., 2024). While this approach transformed previous clunky language models into the friendly assistants we interact with today and was successfully applied to non-LLM contexts (Christiano et al., 2017), it is by no means a final solution for human-AI alignment. More specifically, collecting large amounts of qualitative human feedback is notoriously difficult and resource-intensive. Human annotators are screened based on characteristics such as education level, and then trained or selected to achieve high inter-annotator agreement and expert agreement, such that they agree with each other on how to give specific feedback and also agree with experts (Kreutzer et al., 2018; Chaudhari et al., 2024). The most common two types of feedback used in RLHF are ratings i.e., a given number on a defined scale, and pairwise ranking, i.e., indicating a preferred output from a specific output list. The annotators generally need to ensure LLM outputs align with the triple H (HHH) criteria: Helpfulness, Honesty, and Harmlessness (Askell et al., 2021). The obtained feedback is then further used to train a reward model that will serve as a surrogate for human feedback in further fine-tuning steps. Based on the described constraints, certain limitations of RLHF were observed in recent years (Perez et al., 2022; Casper et al., 2023; Chaudhari et al., 2024). The requirement for large amounts of feedback data is often infeasible and impractical, leading to an imperfect reward model and creating missgeneralization in the context of unseen prompts or situations, where the LLM generates wrong outputs, also known as hallucinations. Another relevant limitation lies in the nature of the delayed human feedback, which is only given at the end of a complete output, which means that the model doesn't receive real-time guidance. Additionally, both the common rating and ranking feedback types are sparse, as they don't communicate to the model the reasoning behind the provided feedback. These and more limitations are elaborated in a recent critical analysis of RLHF (Chaudhari et al., 2024). While the scalability issue is currently being addressed with new methods such as Reinforcement Learning from AI Feedback (RLAIF) (Lee et al., 2023), the lack of density and nuance are yet to be solved.

Implicit feedback, rather than explicit feedback, could address such limitations by extending this approach to training LLM models (Kaufmann et al., 2023). Human communication and interaction with the world is characterized by a richness of non-verbal information in terms of facial expressions, gestures, gaze cues, body movements etc. Some of these non-verbal cues can be decoded through psychophysiological measures such as eye-tracking, heart rate, and electroencephalography (EEG), and could potentially augment or replace explicit feedback to better approximate human preference for training artificial systems (Candon et al., 2023). EEG-based passive brain-computer interfaces (pBCIs) (Zander and Kothe, 2011) have demonstrated the ability to implicitly decipher cognitive and emotional states without the need for user awareness. Among other states, workload (Gerjets et al., 2014; Gherman et al., 2025), surprise (Pawlitzki et al., 2021), and error perception (Parra et al., 2003) have been successfully decoded. PBCIs differ from active and reactive types, as they don't require the user's controlled brain state modulation (Triana-Guzman et al., 2022) or visual stimuli (De Vos et al., 2014) to produce a functioning real-time response in an external system. Instead, the user's naturally occurring mental states to changes in the environment or during the interaction are captured (Zander et al., 2016). Depending on the level of interactivity, this implicit information can either produce open-loop adaptations, where the system responds without altering the user's mental state, or closed-loop adaptations, where the system's response directly influences and modifies the user's mental state (Krol et al., 2018). Systems could gradually learn from this implicit information and adapt to the user in a *neuroadaptive* manner (Zander et al., 2016). While the field of neuroadaptive technology is still an emerging field (Krol and Zander, 2022), recent studies have shown promising results when utilizing EEG-based implicit human rewards in reinforcement learning systems for playing Atari games (Xu et al., 2021) and gamified autonomous driving (Shin et al., 2022). Moreover, the potential of pBCIs for the AI field is gaining increasing media attention (Zander, 2025), and patents for this technology have recently been released (Zander et al., 2024). Using EEG signals in the context of NLP has previously been researched for tasks such as sentiment analysis (Hollenstein et al., 2021) and there exists a large body of literature for averaged event-related potentials (ERPs) for different types of text stimuli (Kaan, 2007), yet pBCI-LLM investigations have not been made so far. In comparison with traditional RLHF methods for LLM alignment, neuroadaptive methods via pBCI integration could meaningfully expand the depth and breadth of user understanding, as non-verbal cognitive and affective reactions to LLM outputs could be captured in real-time, rather than at a delayed pace, hence allowing for more nuance and density in human feedbacks. With our current study, we propose investigating the feasibility of using pBCIs to decode single-trial mental states from text stimuli, as a first step toward neuroadaptive chatbots.

Conforming to the HHH criteria of “harmlessness, honesty, and helpfulness” for LLM output quality, we focus here on two aspects: moral judgment, and error perception. Concerning moral judgment, both cognitive and emotional factors have been found to contribute to a reaction of moral agreement or disagreement with a certain topic or statement (Decety et al., 2012; Hundrieser and Stahl, 2016). The moral stance of an individual can depend on a number of factors such as personality, culture, or motivation (Haidt, 2001) and it has previously been found to activate both prefrontal cortical areas (Fede and Kiehl, 2020), and deeper brain structures (Cunningham et al., 2004). In previous ERP studies, increased potentials such as N400 (Van Berkum et al., 2009) and the Late Positive Potential (LPP) (Van Berkum et al., 2009; Leuthold et al., 2015) have been associated with morally incongruent words, as opposed to neutral or morally-congruent words. In (Van Berkum et al., 2009) pre-selected participants with Christian values were presented with statements such as “I think euthanasia is acceptable/unacceptable” in a word-by-word manner, where the last word in the statement represented the critical event. Similarly, in this study, we will present morally congruent and incongruent statements with a word-by-word approach and critical last words representing our classification trials. With few exceptions, most previous studies looked at averaged neural responses. In (Andreessen, 2023), the data from (Van Berkum et al., 2009) and (Leuthold et al., 2015) have been analyzed at a single-trial level, by training classifiers on morally congruent and incongruent words. The resulting accuracies have not reached significance.

Factual accuracy is always an important criterion used by human annotators to determine the quality of LLM outputs. However, it is difficult to obtain fine-grained information about which specific parts of an output are incorrect (Wu et al., 2023). As such, decoding implicit human reactions of error perception to factual inaccuracy could potentially be useful. When it comes to error perception in the context of text, numerous studies have found an N400 effect associated with the perception of semantic or syntactic errors (Kutas and Hillyard, 1980, 1983; Nieuwland and Van Berkum, 2006). N400 also occurs when expectations of world knowledge are violated (Chwilla and Kolk, 2005; Leuthold et al., 2015; Troyer et al., 2024). For instance, a study compared the averaged EEG signal associated with correct words (“The Dutch trains are *yellow* and very crowded.”), words that are inconsistent with world knowledge (“The Dutch trains are *white* and very crowded.”), and words that should evoke semantic violation (“The Dutch trains are *sour* and very crowded.”) (Hagoort et al., 2004). They found that both semantic and world knowledge violations trigger an N400 effect, but not correct words. Heightened P200 amplitudes when readers encounter either words that violate readers' world knowledge or their moral values have also been observed, indicating early attentional allocation of resources to the processing of unexpected or incongruent information (Leuthold et al., 2015).

In this study, we will test the feasibility of decoding three chatbot-relevant mental states with passive BCI in an offline manner. Firstly, we will examine whether moral salience can be distinguished from neutrality in response to morally charged and neutral words, respectively. Secondly, we will also assess the feasibility of decoding moral judgment by distinguishing reactions to morally congruent and incongruent words. As a strong association has been previously found between affective priming and moral judgment (Decety et al., 2012; Demel et al., 2019), we want to trigger moral reactions more effectively. For this purpose, this study also integrates video-based emotional elicitation with realistic stimuli before sentence presentation. Participants will be selected based on a participant profile with the help of four questionnaires, ensuring the moral judgment paradigm stimuli are relevant and salient. More specifically, the stimuli were related to four topics: immigration, racism, gender equality, and LGBTQ rights, and were meant to induce moral agreement and disagreement. Hence, we administered a battery of four questionnaires before selecting participants. Participants who fit our participant profile had the following trait tendencies: they have a strong preference for quality among social groups, rather than dominance or hierarchy (Pratto et al., 1994); hold less racial prejudices against minorities; have a positive attitude toward homosexuality, and hold no discriminatory attitudes toward women. During the moral judgment paradigm, morally incongruent statements went against these views. Additionally, statements are attributed to fictive moral agents as opposed to more commonly used passive statements, which have also been previously shown to better induce moral reactions (Pantazi, 2012). Lastly, we will assess the feasibility of distinguishing error perception and correctitude perception in reaction to factually incorrect and correct words at a single- trial level, respectively. We interpret error perception here as a reaction to statements that violate factual world knowledge.

As an initial step toward the development of neuroadaptive chatbots, this study aims to answer the following research questions:

1. Can we detect neural correlates of moral salience from human readers at a single-trial level?
2. Can we detect neural correlates of moral agreement and disagreement from human readers at a single-trial level?
3. Can we detect neural correlates of error perception from human readers at a single-trial level?

## 2 Methods

### 2.1 Participants

We administered a battery of four questionnaires before selecting participants. The questionnaires assessed participants' attitudes toward the social justice issues mentioned in Section 1.1. and they were completed digitally: Social Dominance Orientation Questionnaire (SDO-6) (Pratto et al., 1994), Modern Racism Scale (MRS) (Mcconahay, 1986), Attitudes Toward Lesbians and Gay Men (ATLG) (Herek, 1988), and The Ambivalent Sexism Inventory (ASI) (Glick and Fiske, 2018). The wording used in MRS was slightly changed to reflect contemporary terminology by replacing the term “Black people” with “African Americans,” while the rest of the original items were maintained. For all questionnaires, low scores represented a higher tendency to fit our participant profile. Also, participants were asked to rate their level of English proficiency on a scale of 1 (elementary) to 6 (proficient). Based on the obtained response, only participants with English scores of 5 and 6 and a mean of total questionnaire scores under the mean over participants (M = 26.72) were considered. A total of 18 participants responded to our invitation. One participant's data was removed from analyses due to not complying fully with one of the instructions. Other four participants' data were removed from the analysis due to noisy eye-tracking data necessary for the completion of the final stage of this study. The data analyzed in this investigation comes from a total of 13 participants (8 females, 5 males) with a mean age of 30.46 years (SD = 6.60). All invited participants were students at the Brandenburg University of Technology Cottbus Senftenberg.

### 2.2 Procedure

All participants completed a total of three paradigms. The first paradigm consisted of a simulated chatbot interaction where eye-tracking was also recorded. For all participants, this was always the first phase of the experiment and was meant to represent the application task, on which classifiers trained on two calibration paradigms will be applied. The data recorded during this first application phase will not be addressed here, but in an additional study dedicated to the applicability of this pBCI approach. The order of the other two paradigms, the calibration paradigms, was randomized for each participant. In this study, only the data recorded during these calibration paradigms is addressed. These are referred to here as the moral judgment and error-processing paradigms. Written, as well as verbal instructions were provided before each paradigm and a trial version of the corresponding task was presented. The total duration of all the paradigms, including the self-paced breaks, was ~3 h and a half.

### 2.3 Equipment

A total of 64 active actiCAP slim gel electrodes (Brain Products GmbH, Gilching, Germany) were used according to the extended 10–20 international system (Klem et al., 1999). The signal was sampled at 500 Hz with an actiCHamp amplifier. The data was recorded reference-free (where each channel reflects the difference between a single electrode and an internal virtual ground) and average-referenced at the offline analysis stage. The ground electrode was set on the Fpz electrode. During gelling, the electrode impedances were kept under 20 kΩ. Lab Streaming Layer (LSL) (Kothe et al., 2024) was used to synchronize all channel streams.

### 2.4 Paradigms

#### 2.4.1 Moral judgment paradigm

**Figure 1:**
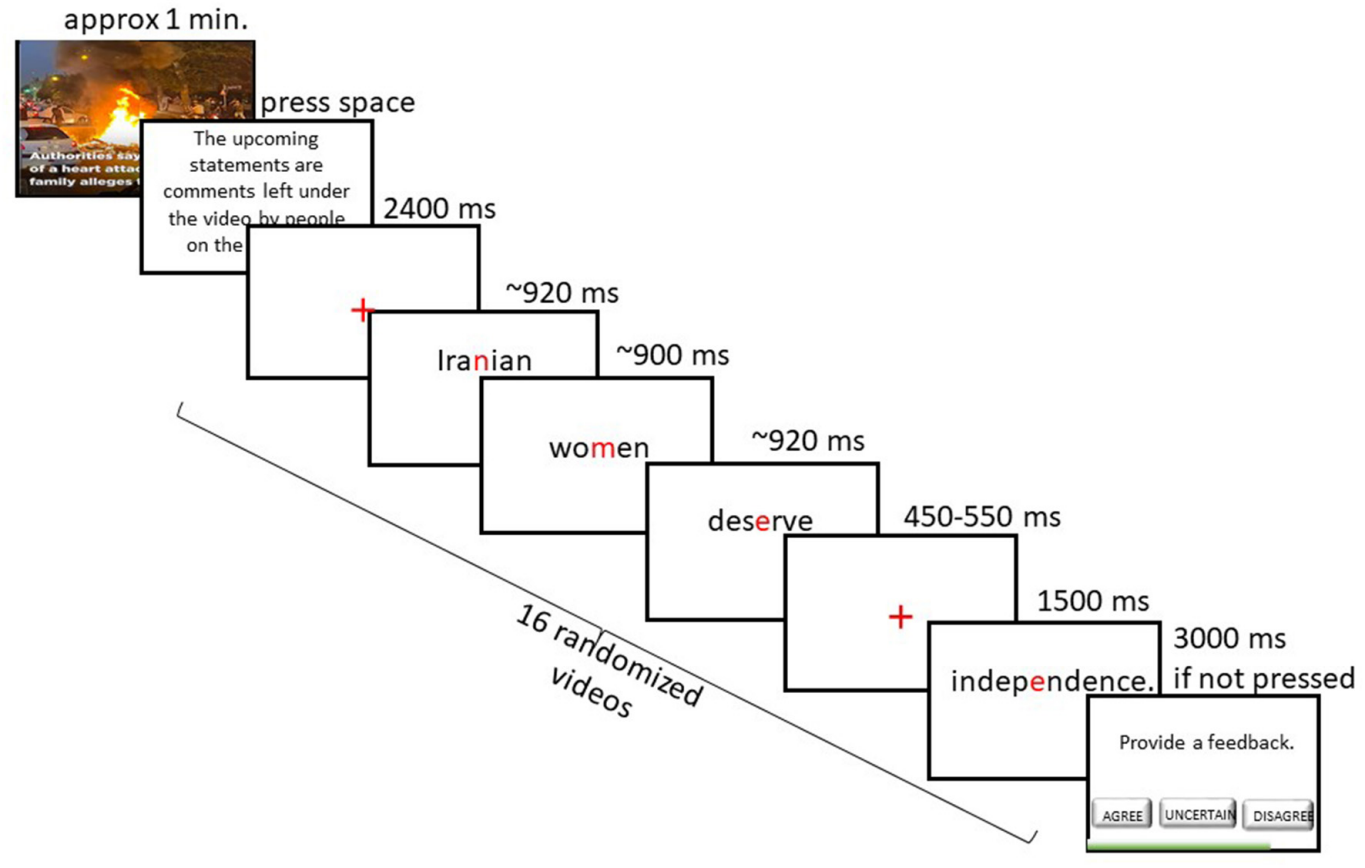
Experimental design for the moral judgment paradigm. The figure illustrates an example^3^ of a morally congruent statement presentation following the presentation of a news video about women's mistreatment in Iran. Participants were instructed to carefully watch each video, then read a series of statements presented word by word, which were meant to represent the comments of other people for the corresponding video. For all statements, the moral stance of each statement would become apparent only when the last word was presented. Then, participants had to indicate by button press if they agreed (left arrow), disagreed (right arrow), or were uncertain (downward arrow) about the read statement with their dominant hand.

The moral judgment paradigm consisted of 16 selected video clips from YouTube^2^, each followed by 10 randomized statements. Each video clip lasted ~1 min and illustrated news pieces of stories around the world related to the four social justice issues. The participants were tasked with carefully watching the videos. Then, statements were presented word by word. We created all statements ourselves as experimenters. To the participants, this was communicated as a selection of comments from YouTube left by strangers on the Internet for the video they had just seen. After each video, an instruction appeared: “You will see a selection of comments from YouTube left by strangers on the Internet for the video you've just seen. Please read each comment carefully and evaluate whether you agree or disagree with the sentiment expressed. Press Enter to continue.” This was disclosed to participants at the end of the experiment. After clicking *Enter*, 10 statements (5 *morally congruent*, 5 *morally incongruent*) related to the seen video were presented in a randomized order. These statements were meant to induce moral agreement and disagreement, in line with the information we gathered in the pretest about this morally aligned group of participants. For example, one video clip showed a news piece detailing the oppression of Iranian women under the Iranian government. Statements associated with this video would either agree with the oppression (“Iranian women not covering their heads should be *imprisoned*.”) or disagree and condemn such oppression (“Women in Iran deserve more *independence*.”). After each statement, participants had 3 s to indicate by a button press if they agree, disagree, or if they are uncertain about the statement they had just read. Participants were also instructed to choose the “uncertain” button whenever they were unsure about the meaning of a word, the general statement, or realized they were not paying enough attention during the sentence presentation. The statements were presented word by word in a Rapid Serial Visualization Presentation (RSVP) (Potter, 2018), with an Optimal Recognition Point (OPR) [also known as the Optimal Viewing Position (OVP)] alignment of words in the center of the screen (Brysbaert and Nazir, 2005), where the aligned characters were colored in red, while the rest of the characters remained black. This display choice was inspired by Spritz™^4^, a speed-reading application that uses the ORP concept to color a key letter within the word at a fixed position, thereby increasing visual focus and reducing eye movements. Previous studies that used this methodology found that text comprehension is not affected by presenting sentences word by word, when compared to traditional reading (Hester et al., 2016). This method ensured a consistent and efficient presentation across trials. The mean number of words per sentence was 8.38, with a standard deviation of 2.37. The last word of each statement served as the critical word, which was either *congruent*, or *incongruent*, representing moral agreement and disagreement, respectively. For all statements, the moral stance of the overall statement was unknown before the presentation of the last word. Before the critical words, a crosshair was presented in the center of the screen at a random presentation time between 450 and 550 ms, introducing a slight variation to avoid predictable timing. The non-critical words were presented for a base duration of 800 ms, with an additional 20 ms for each character beyond the first, as applying a long, fixed duration to these words would have disrupted the natural reading rhythm inherent in the RSVP format. For example, a four-letter word would be presented for 860 ms. The presentation duration for moral target words was fixed at 1,500 ms, irrespective of word length, to ensure that participants had sufficient time to fully process the moral meaning of each word in relation to the sentence context and accompanying video stimuli. As these words dictated the moral stance of the entire sentence, a consistent and extended presentation time was essential to support deep cognitive engagement. This design ensured both sufficient moral processing time and a smooth reading experience for non-moral content. Between the statement assessment and the next statement, a crosshair was presented in the middle of the screen for 2,400 ms to induce a mental break between statements. In total, there were 160 balanced statements and hence, 80 *morally congruent* and 80 *morally incongruent* trials. A schematic illustration of this paradigm is shown in Figure 1. A total of 4 self-paced breaks were introduced. To investigate the effectiveness of our affective priming approach, the Positive and Negative Affect Schedule (PANAS) (Watson et al., 1988) questionnaire was administered before and after completing the paradigm. This questionnaire is meant to measure the positive and negative affect at a given time. Only the scores for the items “distressed” and “upset” were statistically analyzed here.

#### 2.4.2 Error-processing paradigm

Participants saw the following instruction at the beginning of the task: “Review the following statements and assess their correctness. Press Enter to continue.” After pressing *Enter*, the instruction did not repeat. Statements were presented with the same RSVP-OPR approach and the same speed as the moral judgment paradigm. No video stimuli were present in this paradigm. Hence, the non-critical words were presented at the pace of 800 ms basis and 20 ms extra for each character besides the first one. Critical last words would dictate the correctness of the entire statement and were all presented for 1,500 ms. Before each of these critical words, a crosshair was also presented in the middle of the screen at a random speed between 450 and 550 ms. Between statements, a crosshair was presented for 2,400 ms to allow a mental break. After each statement presentation, participants had 3 s to indicate through a keyboard button if they thought the statements were correct (left arrow), incorrect (right arrow), or were uncertain about their correctness (downward arrow). Also, participants were instructed to choose the “uncertain” button whenever they were unsure about the meaning of a particular word, the general statement, or realized they were not paying enough attention. The statements focus on obvious world knowledge regarding topics such as geography, culture and language, food, or basic facts of science and were either wrong (“A baby cat is called a *puppy*.”) or correct (“The taste of sugar is *sweet*.”). A total of 160 general statements (80 *correct*, 80 *incorrect*) were created by experimenters.

### 2.5 EEG processing

The EEG processing EEGLAB v2021.0 (Delorme and Makeig, 2004) was used to pre-process data. The EEG data for all participants and both paradigms went through a few pre-processing steps in preparation for independent component analysis (ICA) decomposition with an AMICA algorithm (Palmer et al., 2011). The non-experimental data, such as data recorded during breaks was removed. The data was resampled to 250 Hz. The EEGLAB function *clean_artifacts* was used to remove noisy channels and filter the data with a FIR forward-backward Kaiser filter at a 0.5 Hz cutoff edge. Channels with a correlation below 0.8 to a robust estimate or exhibiting line-noise outliers above 4 standard deviations were removed, while flatline, burst, and window criteria were disabled to focus on channel-level noise. A spherical interpolation method was applied afterward. Then, channels were re-referenced to a full-rank common average reference. Finally, the noise-cleaned data was passed to the AMICA algorithm with automatic sample rejection parameters (Klug et al., 2022). We set the following rejection parameters: *do_reject* = 1 (rejects the outliers for the model being computed), *numrej* = 5 (five rounds of outlier rejection), and *rejsig* = 3 (flag samples falling more than three standard deviations below the model likelihood). After independent components were obtained, these were labeled with the ICLabel algorithm version 1.4 with default parameters (Pion-Tonachini et al., 2019). The EEGLAB DIPFIT plugin was used for dipole fitting.

### 2.6 EEG classification

#### 2.6.1 General classification method

A series of classification investigations were performed. The following steps are common to all classification types. All models were trained in an offline manner, using MATLAB R2022a (The Mathworks, Inc., Natick, MA, USA) and BCILAB 1.4-devel (Kothe and Makeig, 2013). For each participant, the models were trained on 80% of the data and tested on 20% of each corresponding dataset, referred to in the following sections as training data and testing data, respectively. We chose this approach to mimic a realistic pBCI setup, where a classifier is initially calibrated for individual participants and tasks and then applied online. To investigate the validity of the underlying neural signal used by classification models, all classifications were performed twice: firstly without removing artifact components, and then by removing non-brain components with 15% residual variance after the automatic ICALabel labeling. We refer to these two versions of data as *full-component* data and *brain-component* data. Before classification, trials that did not conform to the ground labels were removed from training for both paradigms. We will refer to these trials as *inconsistent trials* going forward. For example, if the ground label of a specific trial in the moral judgment paradigm was set to *morally congruent*, but the participant indicated *disagree* or *uncertain*, this trial was not included in the training and testing of the classification model. Similarly, if the ground label of a specific trial in the correctness paradigm was set to *correct*, but the participant indicated *incorrect* or *uncertain*, this trial was removed from the classification. On average, 16.85 trials (SD = 11.42) per participant were removed in the moral congruence analysis and 9.15 (SD = 4.08) trials were removed in the error-processing analysis. Across all participants, this represented ~10.53% of the total moral congruence trials, and 5.71% of the total correctness trials, respectively. For all classifications, a windowed means approach was used to extract features, which uses the averaged potential amplitude (Blankertz et al., 2011) obtained from non-normalized data. Epochs of 1 s were extracted at stimulus onset in each case. The chosen time windows for ERP feature extraction were based on both prior literature and inspection of the grand-averaged data. A 200–800 ms interval was selected to focus on the ERPs of interest, mainly the N400 (300–500 ms), P600 (500–700 ms), and LPP (500–800 ms). A limit of 200 ms was set to exclude early sensory responses (e.g., P1, N1), which are less relevant for the investigated mental states. For all classifications, the data was bandpass-filtered between 0.1 and 15 Hz, and the training was done with a regularized linear discriminant analysis (LDA) with a 5-fold cross-validation. We chose LDA, as it has been regarded as a highly robust and popular algorithm for BCI classification, exceeding performance obtained with more complex algorithms (Lotte et al., 2018). The features were extracted from all 64 EEG channels without prior spatial selection. This allowed the classifier to utilize the full spatial information available and identify the most discriminative electrodes for each participant and condition.

#### 2.6.2 Moral vs. neutral

To investigate the feasibility of detecting neural correlates of moral salience, a classification model was trained and tested to distinguish *moral* vs. *neutral* classes with the approach described above. After the inconsistent trials were removed, the morally congruent and morally incongruent trials were combined in a single class by renaming them as *moral*. To obtain the neutral class, a selection of non-critical words present in the statements was renamed to *neutral*. Some examples of selected neutral words are: “should, generally, about, concept, idea, fact.” After the trial numbers were balanced in number, a total of 90 trials per class resulted. Then, the cross-validated training was performed on 80% of these trials, and tested on 20% of the data. The windowed means approach used 12 sets of 50 ms time windows between 200 and 800 ms. To account for variability in randomized trial selection, the classification was performed 10 times per data type (full-component data, brain-component data, as previously described).

#### 2.6.3 Morally congruent vs. morally incongruent

The same datasets recorded during the moral judgment paradigm were used for this classification, which we refer to as moral congruence classification. Again, the inconsistent trials were removed, and then the training and testing of a classification model for *morally congruent* and *morally incongruent* classes was performed. The windowed means approach used 12 sets of 50 ms time windows between 200 and 800 ms. Again, this classification was done twice, with full components in and non-brain components removed.

#### 2.6.4 Correct vs. incorrect

The datasets recorded during the error processing paradigm were used for this classification. Firstly, the inconsistent trials were removed. Then, the training and testing were performed for correct and incorrect classes using the 12 sets of 50 ms time windows between 200 and 800 ms. The classification was performed twice, with and without removing non-brain components.

## 3 Results

### 3.1 Event-related potentials

**Figure 2:**
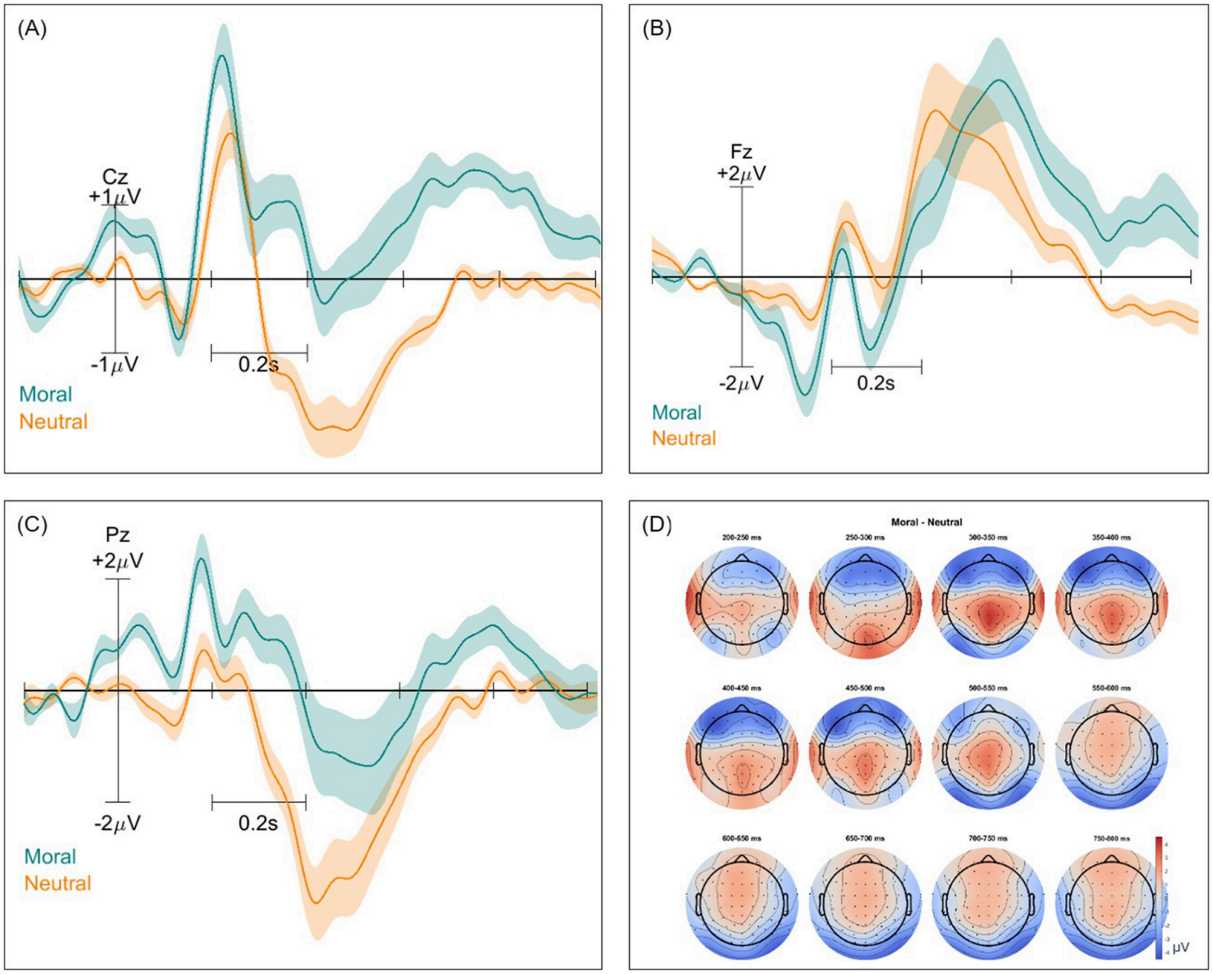
Event-related potentials (ERPs) and topographical maps illustrating neural responses to morally charged and neutral stimuli for full-component data. **(A–C)** Grand-averaged ERP waveforms recorded at electrodes Cz, Fz, and Pz, with shaded areas representing standard errors. Morally-charged trials are shown in blue, while neutral trials are in orange. **(D)** Scalp topographies of ERP differences over 12 time windows between 200 and 800 ms, highlighting differences in neural activation patterns across the scalp.

**Figure 3:**
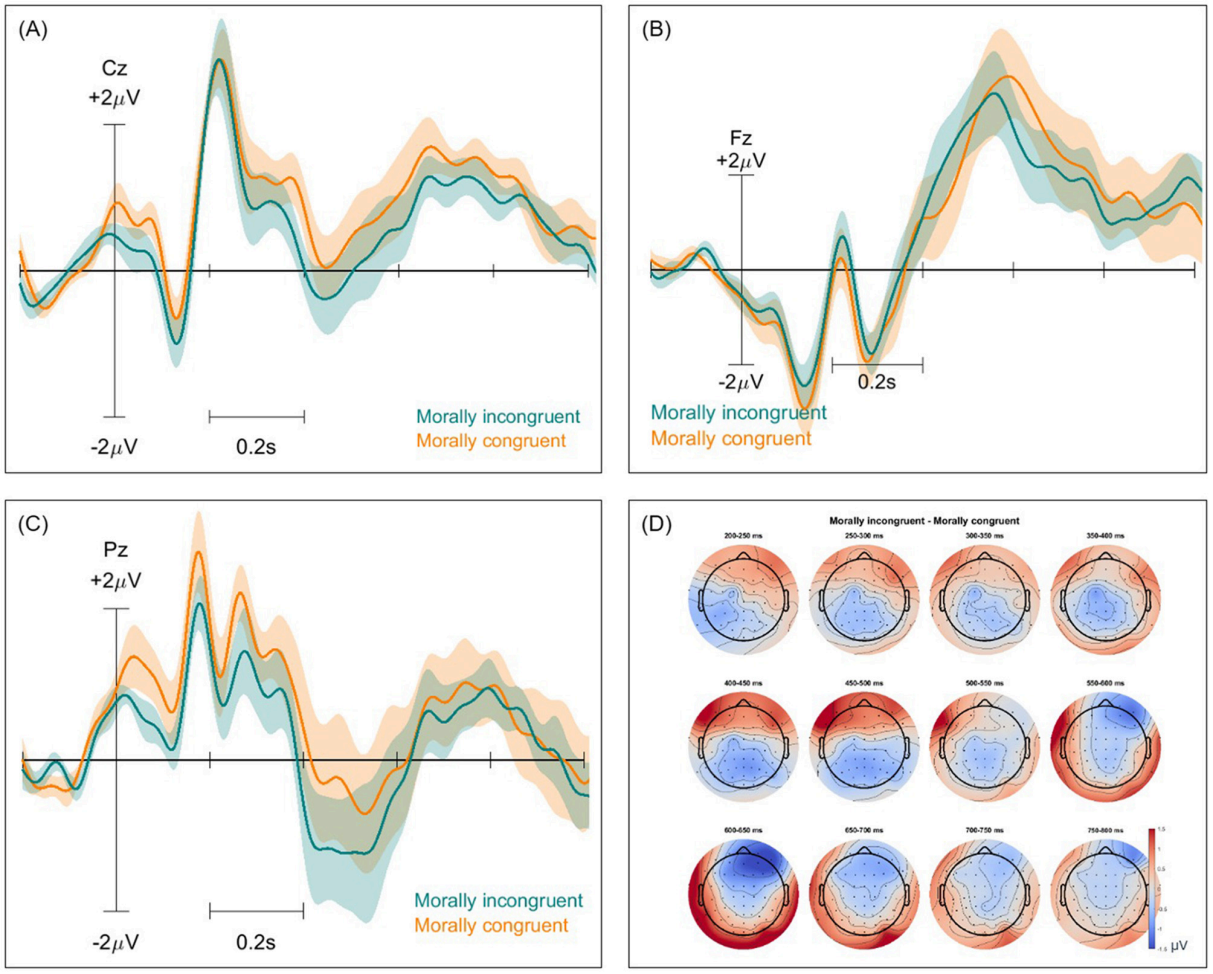
Event-related potentials (ERPs) and topographical maps illustrating neural responses to morally incongruent and congruent stimuli for full-component data. **(A–C)** Grand-averaged ERP waveforms recorded at electrodes Cz, Fz, and Pz, with shaded areas representing standard errors. Morally incongruent trials are shown in blue, while morally congruent trials are in orange. **(D)** Scalp topographies of ERP differences over 12 time windows between 200 and 800 ms, highlighting differences in neural activation patterns across the scalp.

**Figure 4:**
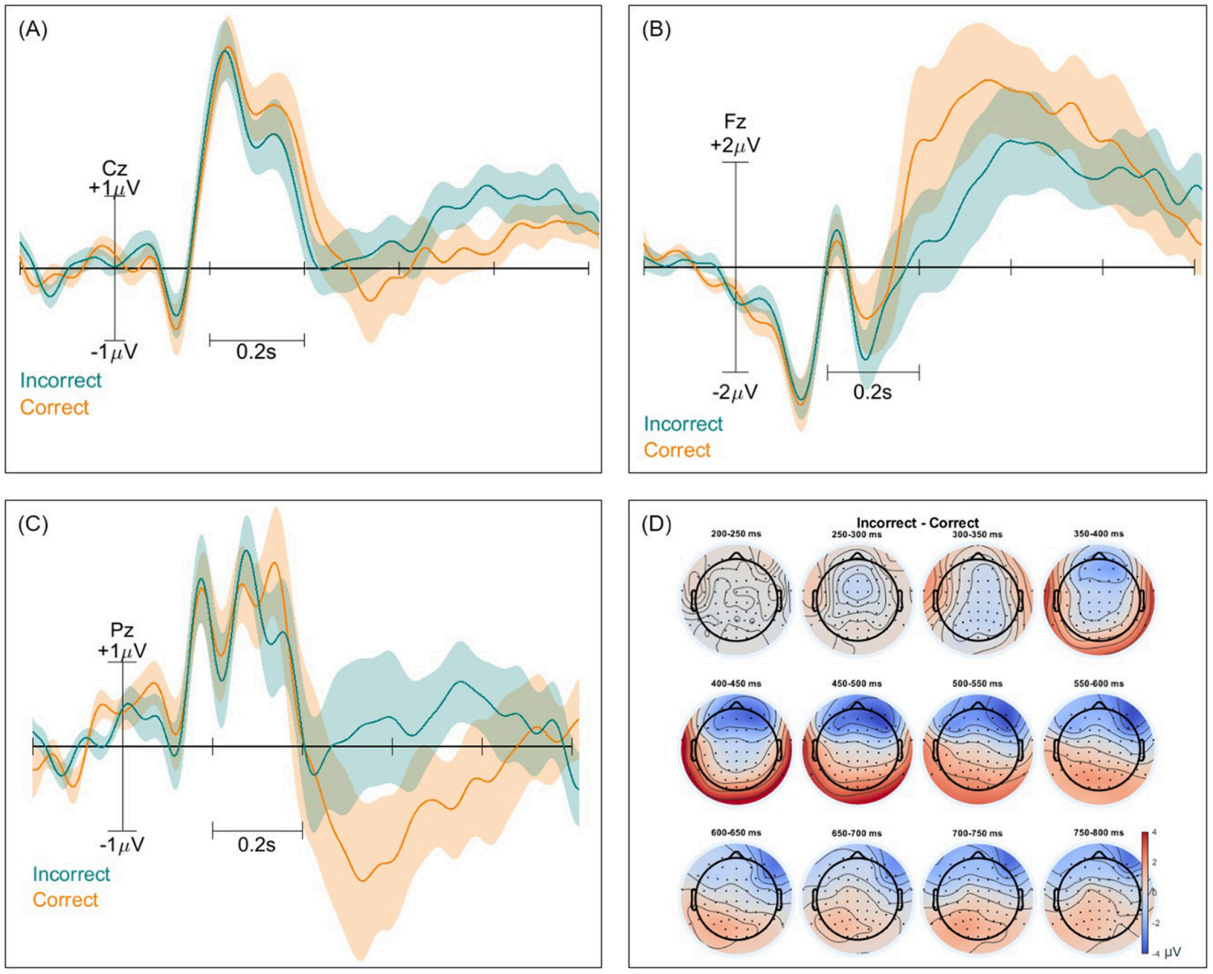
Event-related potentials (ERPs) and topographical maps illustrating neural responses to incorrect and correct stimuli for full-component data. **(A–C)** Grand-averaged ERP waveforms recorded at electrodes Cz, Fz, and Pz, with shaded areas representing standard errors. Incorrect trials are shown in blue, while correct trials are in orange. **(D)** Scalp topographies of ERPs differences over 12 time windows between 200 and 800 ms for both conditions, highlighting differences in neural activation patterns across the scalp.

Event-related potentials (ERPs) and ERP difference scalp maps were inspected for both paradigms and all classification types. These were computed twice for each pair of classes, with and without the removal of non-brain components. Figures 2–4 show these ERPs for the moral vs. neutral, morally congruent and morally incongruent, and correct vs. incorrect class pairs, respectively. We showcase here grand-averaged ERP plots for channels Fz, Cz, and Pz for full component data. Additional ERP plots for the same channels, derived from data containing only brain components, are available in the Supplementary Figures 1–3. It can be observed that both full-component and brain-component data produced very similar neural signals in all instances, validating that our classifiers use the signal information generated at the cortical level to distinguish classes. In all cases, a 0.1 Hz high pass filter, and a 15 Hz low pass filter were applied. A baseline of −200 to 0 was used to obtain all ERPs.

### 3.2 Classification results and statistical results

The training and testing average classification accuracy results obtained for all the described classification models are outlined in Table 1. The shown average classification results for the moral vs. neutral classification analysis were obtained after averaging accuracy results within subjects for the 10 classification runs and then averaging these values across subjects. The average standard deviation over classification runs across training accuracies was 3% accuracy points for full-component data and 3% accuracy points for brain-component data. The average standard deviation over classification runs across test accuracies was 5% accuracy points for full-component data, and 5% accuracy points for brain-component data. Individual subject classification accuracies for full-component and brain-component data for all classification analyses can be found in the Supplementary Figures 4, 5. For all analyses, the chance accuracy was computed by simulating a random classifier that guessed labels in proportion to the observed class frequencies and taking the upper bound of its one-sided 95% Wilson confidence interval (Billinger et al., 2013; Müller-Putz et al., 2008). For the moral salience classification (moral vs. neutral), we obtained training results significantly above chance for all participants and classification runs for the full-component data. We obtained training results significantly above chance for all participants across 8 classification runs, while one participant did not achieve significance for 2 of the 10 classification runs for brain-component data. The moral congruence (morally congruent vs. morally incongruent) classification led to training classification results significantly above chance for 1 out of the 13 participants for the full-component data, and 2 out of the 13 participants for the brain-component data. We obtained significantly above-chance training results for the error-processing (correct vs. incorrect) classification for 12 out of 13 participants for the full-component data and 10 out of 13 participants for the brain-component data. For each subject and each classification analysis, the activation patterns of the classification were also obtained by multiplying corresponding feature covariances and classifier weights, in line with the method proposed by Haufe et al. (2014). The average group-level patterns can be found in the Supplementary Figures 6–11 for classification analyses on both full-component and brain-component data types. These activation patterns illustrate the contributions of individual features to the discriminative signal.

**Table 1:** Training and testing mean classification results across conditions and component types.

**Classification type**	**Components**	**Training accuracy Mean (%)**	**Training accuracy SD (%)**	**Testing accuracy Mean (%)**	**Testing accuracy SD (%)**
moral vs. neutral	all	78	6	76	8
moral vs. neutral	brain	76	6	74	11
morally congruent vs. morally incongruent	all	49	6	52	9
morally congruent vs. morally incongruent	brain	50	6	52	10
correct vs. incorrect	all	66	8	62	10
correct vs. incorrect	brain	63	6	59	9

### 3.3 Affective priming results

One one-tailed paired *t*-test revealed the “distressed” scores after completing the moral judgment paradigm (M = 2.33, SD = 1.30) were marginally significantly higher than the “distressed” scores before completing the paradigm (M = 1.66, SD = 0.89) (*p* = 0.08). Another one-tailed paired *t*-test yielded marginally significantly higher scores obtained for the “upset” item after the moral judgment paradigm (M = 2.08, SD = 1.24) than the scores obtained before completing the paradigm (M = 1.42, SD = 0.90) (*p* = 0.09). One subject was excluded from this analysis, as the corresponding scores for the investigated scales before completing the paradigm were missing.

## 4 Discussion

### 4.1 Mental states decoding

In this study, we investigated the detection of neural correlates of moral salience, moral judgment and error processing from human readers at a single-trial level. Our efforts represent an initial step toward a better understanding of the feasibility of pBCI-enabled implicit human feedback for LLMs. For this purpose, we recorded EEG data from 13 participants who completed two reading paradigms. In both these paradigms, statements were presented and read word by word, in an RSVP manner with an OPR alignment. For the moral judgment paradigm, video-based affective priming was also included before presenting the statements. With our approach, we were able to successfully distinguish moral salience from text stimuli, as compared to neutral stimuli. Our results were not as encouraging for moral judgment decoding, where we obtained chance-level results. More specifically, we demonstrate the feasibility of classifying single-trial reactions to morally-charged words, but not the ability to differentiate between moral agreement and disagreement. Similar chance-level results were obtained in (Andreessen, 2023), where classification on data from (Van Berkum et al., 2009) and (Leuthold et al., 2015) was investigated at a single-trial level for reactions to morally congruent and morally incongruent words. The low classification performance is not surprising given the hardly distinguishable difference in ERP waveforms between morally congruent and morally incongruent trials. Hence, affective priming with realistic video-based stimuli before statement presentation did not make a difference in our case, although we achieved marginally significant negative affective priming effects according to our questionnaire results. We obtained moderate classification performance during calibration for both full-component and brain-only data, for the error-processing classification. Still, a drop in accuracy was observed when applying the cross-validated models to the test data.

Our results hint toward the potential feasibility of accessing key mental states at a single-trial level from just a few milliseconds of data in reaction to text stimuli. While more work is needed to increase decoding performance, we can envision what it would mean to include pBCI-enabled implicit human feedback during the training of LLMs, or during user interaction after deployment. If this integration indeed becomes possible, we can speculate that LLMs become implicitly aware of the moral saliency of an ongoing interaction, perhaps learning that particular topics are sensitive for the user and that a change of tone might be appropriate. In time, this deeper insight into the moral sensitivities of the user could nurture a more intimate, personalized alliance between the man and machine. Moreover, gaining a nuanced understanding of what an annotator considers to be factually correct or incorrect, could improve the RLHF scaling by providing more human feedback data, thereby reducing the chance for hallucination in future deployed models (Huang et al., 2024).

### 4.2 ERP signatures

The ERP morphologies for the investigated mental states resembled those reported in ERP studies of moral reactions. We observed an increased P200 in the centroparietal region for the morally charged words, which aligns with other findings (Chen et al., 2009; Leuthold et al., 2015). A small P200 increase can be observed for the morally incongruent words in the frontal region, as compared to morally congruent words, in line with previous investigations (Hundrieser et al., 2021; Gao et al., 2023). This suggests an early automatic moral salience detection due to attentional allocation, confirming a potential “moral pop-out effect,” where moral words are more perceptually salient (Gantman et al., 2020). A broad positivity in the ERP for morally charged words, as compared to neutral words could also be observed between 400 and 800 ms, which we attribute to the LPP effect. Similarly, in a study that aimed to uncover the time course of moral perception, a comparison was made between moral words and non-moral words. The researchers found a significantly larger LPP for moral words, even when controlling for arousal and emotional valence (Gantman et al., 2020). This effect is usually interpreted to signify an increase in the attentional allocation resources. Unlike findings from (Leuthold et al., 2015) and (Van Berkum et al., 2009), who reported a larger positivity for morally unacceptable words between 500 and 1,000 ms, we did not observe the same effect for the ERP corresponding to morally incongruent vs. congruent words. However, we did observe a more negative potential for morally incongruent words in the central-parietal region, which may reflect the N400 effect found in these and other studies (Hundrieser and Stahl, 2016).

Interestingly, we could not observe an N400 effect for the world knowledge violation trials. This differs from other studies on factual violation that did identify an N400 effect for the centro-parietal region (Hagoort et al., 2004; Leuthold et al., 2015). The absence of an N400 could potentially be explained by the fact that none of our participants were English native speakers, the language used to create our text stimuli, although only participants with a high level of self-reported English proficiency were included in the study. It has been previously found that bilingual readers might not be able to predict sentence-final words in their second language in the same manner as natives, failing to show an N400 (Martin et al., 2013). Instead, a broad positivity could be observed for incorrect trials in this scalp region after 500 ms, which we attribute to the so-called “semantic P600” (van Herten et al., 2005). While traditionally associated with syntactic anomalies, many studies have recently observed a positive deflection peaking at around 600 ms for semantic anomalies as well (Zheng and Lemhöfer, 2019; Seyednozadi et al., 2021), which we believe we can observe in the centroparietal region in our ERP plots. While our focus in this study was single-trial classification, the ERP signatures we uncovered suggest the validity of the mental state elicitation. Additionally, these findings offer more insights into the expected neural patterns for LLM-relevant mental states at the individual world level. It remains to be further investigated what specific neural signals are elicited during chatbot interactions, for larger snippets of text.

### 4.3 Limitations and future directions

The results we obtained here are promising, as they lay the groundwork for enabling implicit human feedback for LLMs. Nevertheless, several limitations in our study should be mentioned. Firstly, we based our classification for moral salience on combined morally congruent and incongruent trials, as compared to selected neutral words. However, the neutral words are embedded within the statements, in contrast with the morally charged trials, which appear after a fixation cross as ending words in the statements. While we chose a broad and later time window for the classification of moral salience, other studies should also explore classification on word stimuli presented invariably. Besides the positioning within the sentence, moral and neutral words also differ in terms of semantic salience, as in contrast to the neutral words, the moral words offer semantic meaning to the sentences, which was intentionally designed here to isolate the potential moral reactions of readers. Moral words also naturally carry greater emotional valence and arousal in comparison to neutral words (Marques et al., 2022). Thus, our analysis reflects a broader cognitive response to morally charged words vs. neutral words, without attributing the observed effect solely to the moral dimension. From this point of view, the morally congruent vs. morally incongruent classification can be seen as a more direct test of the moral processes.

Moreover, similar investigations should explore pBCI classification on moral and error-processing elicited from text without the requirement for explicit feedback. Given the preliminary nature of this investigation, we chose to gain complete knowledge regarding the subjective processing of the constructed sentences, such that we can remove from the calibration process trials that do not conform to the ground labels of classes. Still, previous studies have found potential differences in neural activations and cognitive functions between implicit and explicit moral reasoning (Greene et al., 2004; Fede and Kiehl, 2020), which should be addressed.

Lastly, with our study, we tried to mimic a realistic BCI scenario, where calibrated classifiers are applied in an online setting by keeping a portion of our data as test data. More research is needed to understand if pBCI classifiers can be successfully applied in the context of chatbot interfaces where text is being read not word by word, but word after word. For such applications, eye-tracking is needed to match the gaze of the reader with the corresponding text snippets that elicit specific mental states. Building on these promising findings, we plan to investigate the integration of eye-tracking and pBCI classification in a simulated chatbot setting, bringing us closer to real-life applications of implicit human feedback. This outlook is elaborated in Gherman and Zander (2024). While two mental states are discussed in this paper, one can imagine that others, such as confusion, cognitive workload, and surprise would also be relevant. As such, decoding of multiple mental states could happen in parallel, while an eye-tracker detects the words or text snipes that are being read. For instance, once high confusion and high cognitive workload are decoded via pBCI in reaction to a given output (e.g., an overview of quantum mechanics) and concomitantly inform the LLM system of this change, the LLM could follow up by offering an alternative, simpler explanation on a given sub-topic that triggered these mental states (e.g., quantum entanglement). While significant progress in sensor technology that is more compatible with real life has been made in recent years (Niso et al., 2023), there are still major leaps required before such sensors can be robustly worn by humans tasked to supervise AI systems. Moreover, more work needs to be done on the software side to achieve universal classification, as BCI systems used today still require a lengthy calibration phase for each task, subject, and electrode type. Additionally, even if such obstacles toward realistic uses of BCI are overcome, it is currently unclear how to safely navigate regulatory constraints for mental state detection via automated tools [European Union, 2024, Article 5(1)(f)], while strictly maintaining and safeguarding the privacy of users.

## 5 Conclusion

This investigation explored the feasibility of mental state decoding from text stimuli at a single-trial level. While more validation is needed, our findings suggest that moral salience and error processing might be inferred from single-trial data with passive BCIs. Further distinguishing between moral agreement and disagreement in reaction to morally congruent and incongruent words presented a challenge. The obtained ERP patterns partly confirmed successful elicitation of the investigated mental states and aligned with some of the previous neuroscientific findings. Going forward, we plan to investigate mental state classification in more realistic, chatbot-like scenarios. Taken together, our results hint toward the possibility of accessing human implicit feedback through passive BCIs, which could complement current AI training methods. Moreover, more human nuance and a better understanding of human values could be provided during chatbot interactions if this implicit channel of communication becomes available. With our work, we uncover a potential novel path toward better alignment of LLMs and AI models in general through the use of passively decoded implicit human feedback.

## Data Availability

The raw data supporting the conclusions of this article will be made available by the authors, without undue reservation.

## Footnotes

^1^ www.chatgpt.com

^2^ www.youtube.com

^3^ https://youtube.com/shorts/5MiYDE8y1VI?si=CWXvE1b3tgh3e8i4

^4^ www.spritz.com
