# Supplementary material for: Towards neuroadaptive chatbots: a feasibility study
Source: Front Neuroergon. 2025 Oct 15;6:1589734. doi: 10.3389/fnrgo.2025.1589734 (PMC12568581; doi:10.3389/fnrgo.2025.1589734)
Supplement: Supplementary file 1 [file Data_Sheet_1.docx]

Supplementary Material

**
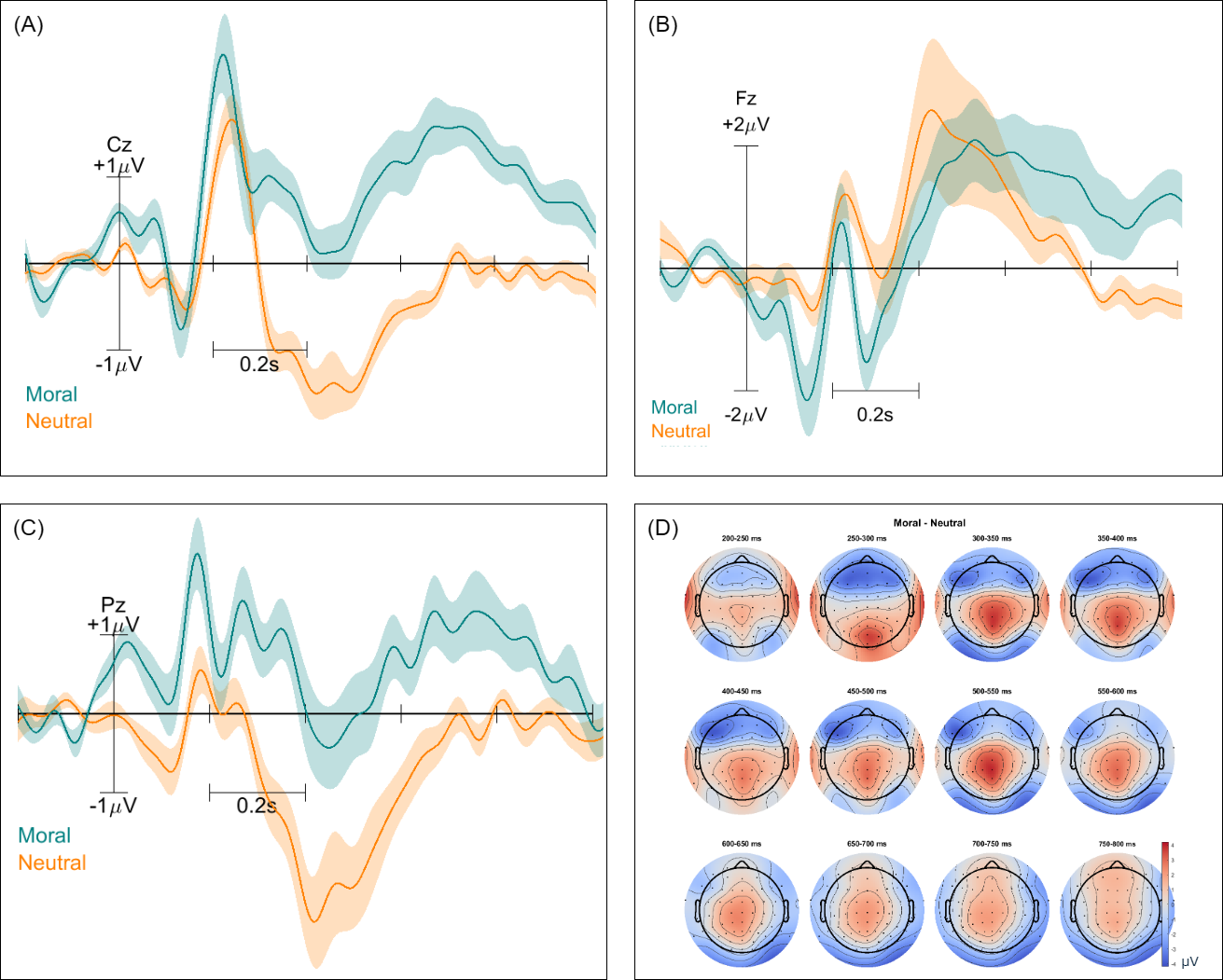
**

**Supplementary Figure 1.** Event-related potentials (ERPs) and topographical maps illustrating neural responses to morally charged and neutral stimuli for brain-component data. (A-C) Grand-averaged ERP waveforms recorded at electrodes Cz, Fz, and Pz, with shaded areas representing standard errors. Morally-charged trials are shown in blue, while neutral trials are in orange. (D) Scalp topographies of ERP differences over 12 time windows between 200-800 ms, highlighting differences in neural activation patterns across the scalp.


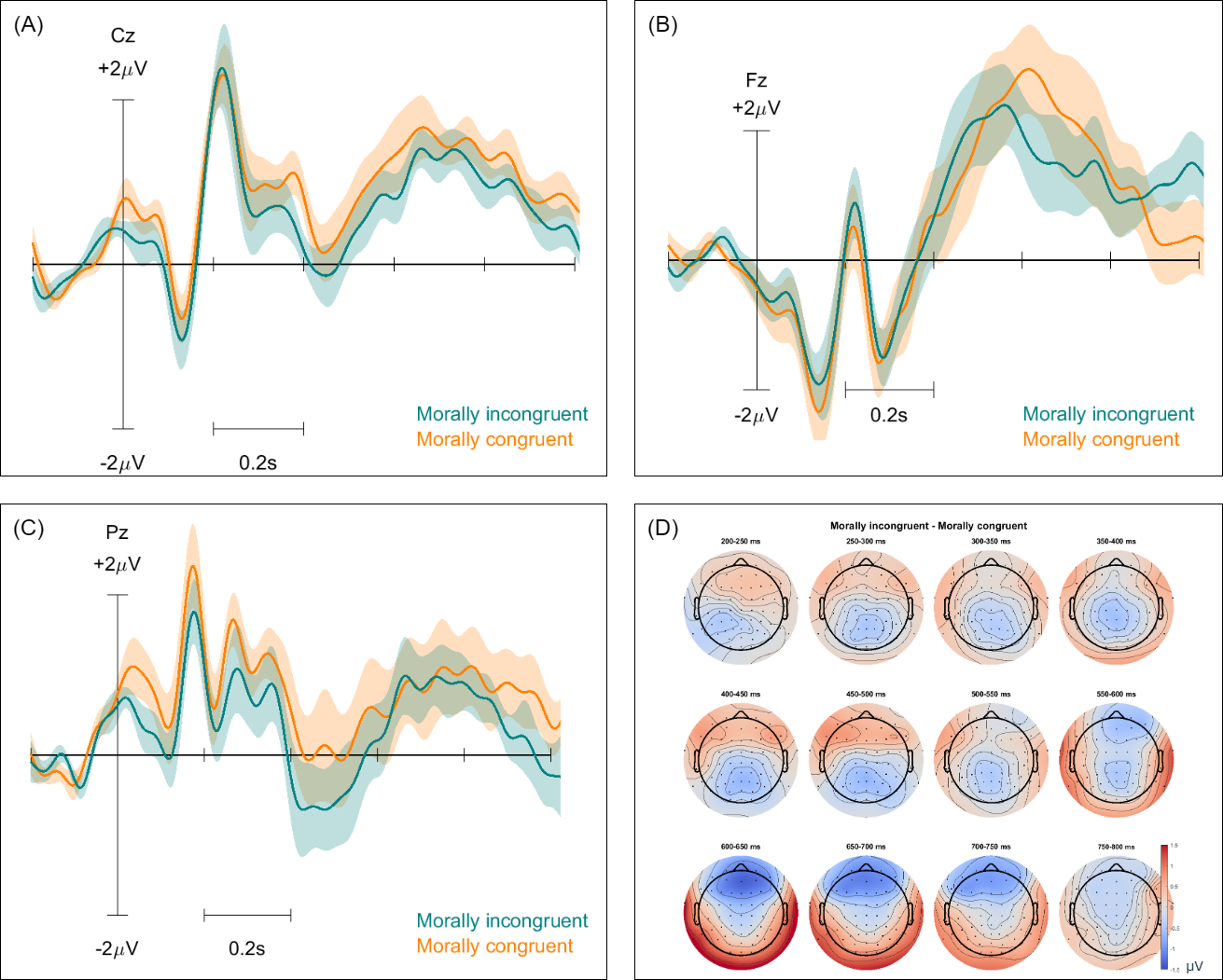


**Supplementary Figure 2.** Event-related potentials (ERPs) and topographical maps illustrating neural responses to morally incongruent and congruent stimuli for brain-component data. (A-C) Grand-averaged ERP waveforms recorded at electrodes Cz, Fz, and Pz, with shaded areas representing standard errors. Morally incongruent trials are shown in blue, while morally congruent trials are in orange. (D) Scalp topographies of ERP differences over 12 time windows between 200-800 for both conditions, highlighting differences in neural activation patterns across the scalp.


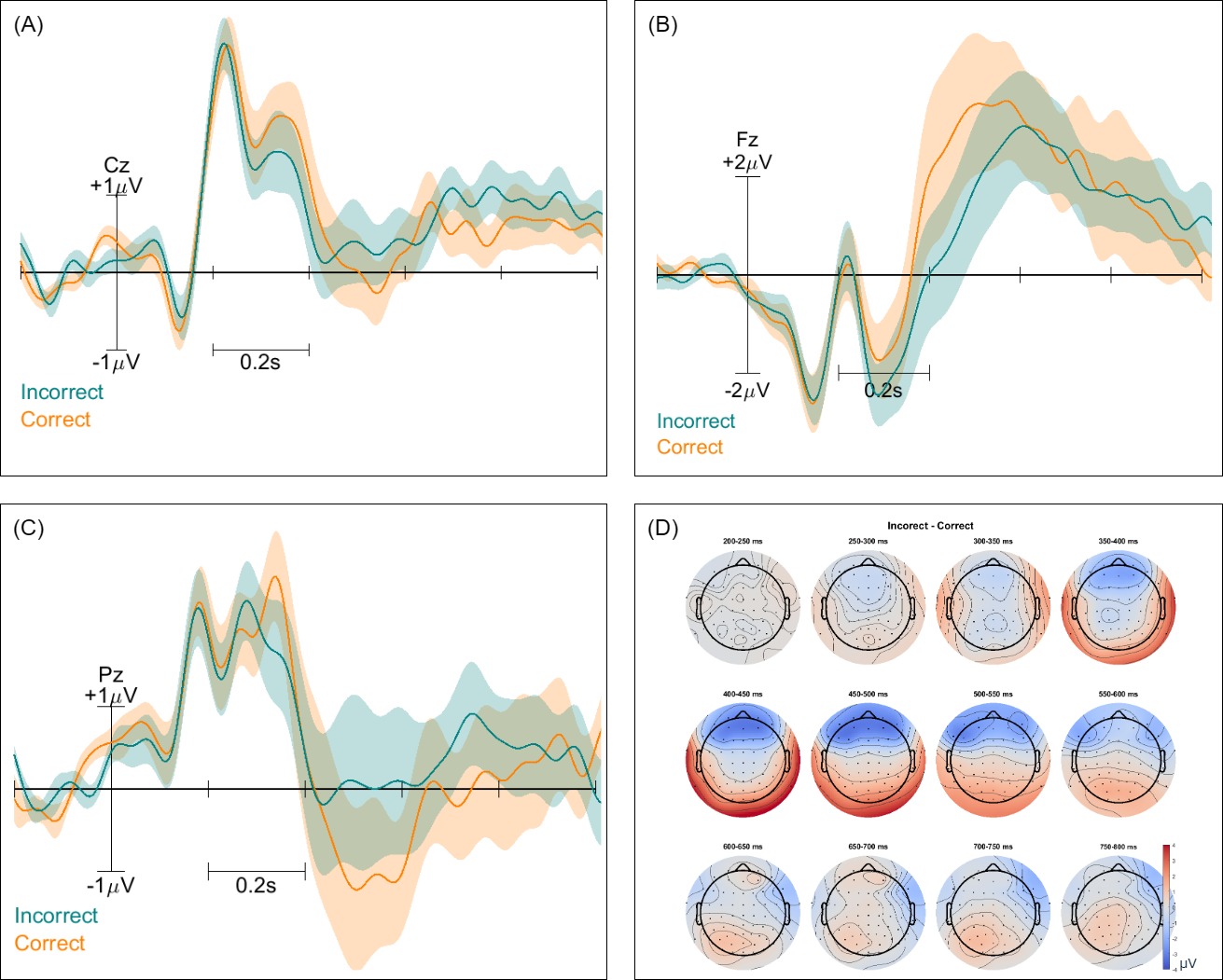


**Supplementary Figure 3.** Event-related potentials (ERPs) and topographical maps illustrating neural responses to incorrect and correct stimuli for brain-component data. (A-C) Grand-averaged ERP waveforms recorded at electrodes Cz, Fz, and Pz, with shaded areas representing standard errors. Incorrect trials are shown in blue, while correct trials are in orange. (D) Scalp topographies of ERP differences over 12 time windows between 200-800, highlighting differences in neural activation patterns across the scalp.

**
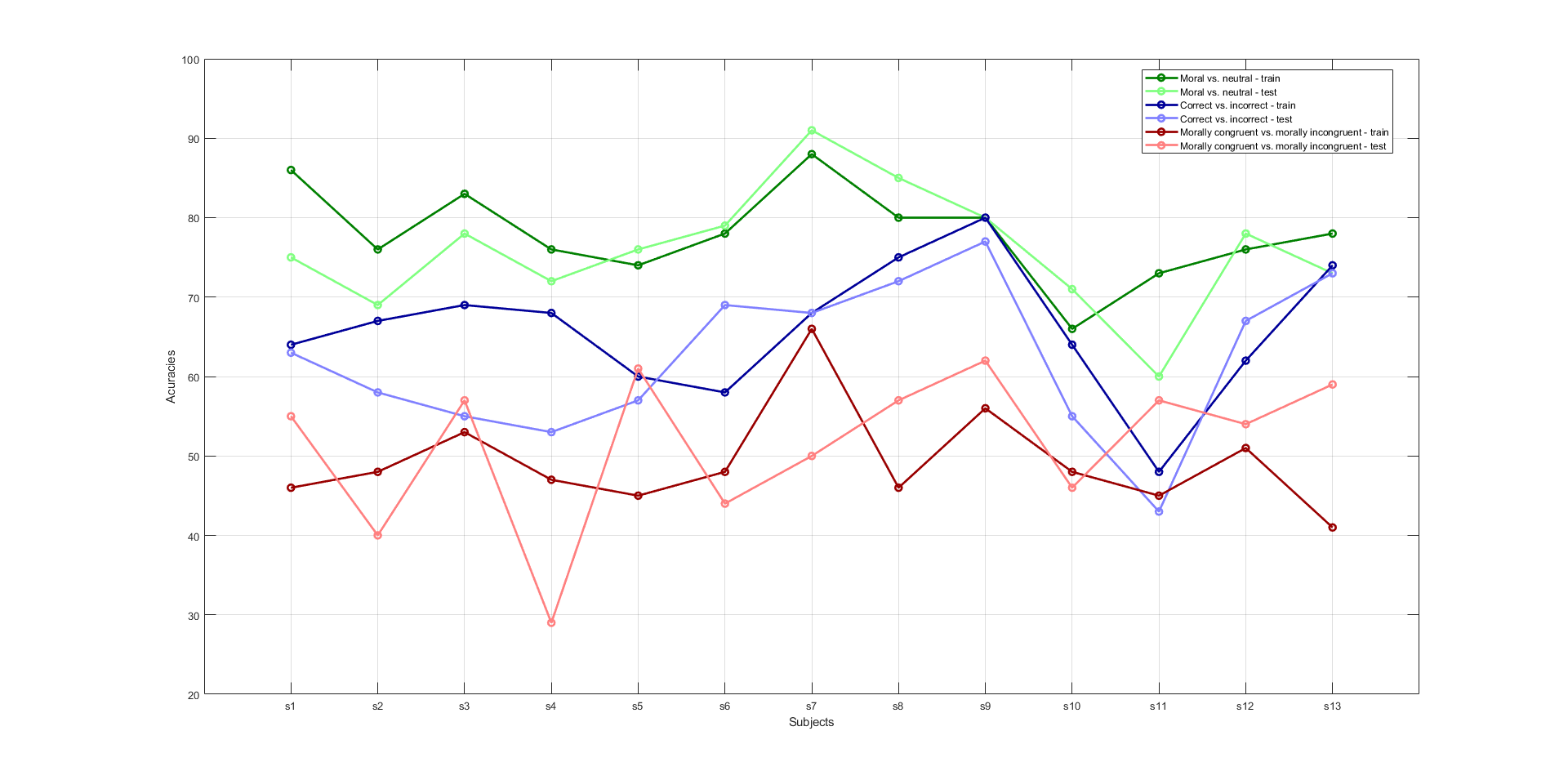
Supplementary Figure 4.** Training and testing classification accuracies across individual subjects obtained on full-component data for each of the three classification analyses: moral versus neutral (green), morally congruent versus morally incongruent (red), and correct versus incorrect (blue). The individual results shown for the moral versus neutral classification reflect the average accuracy per subject, computed across 10 classification runs.


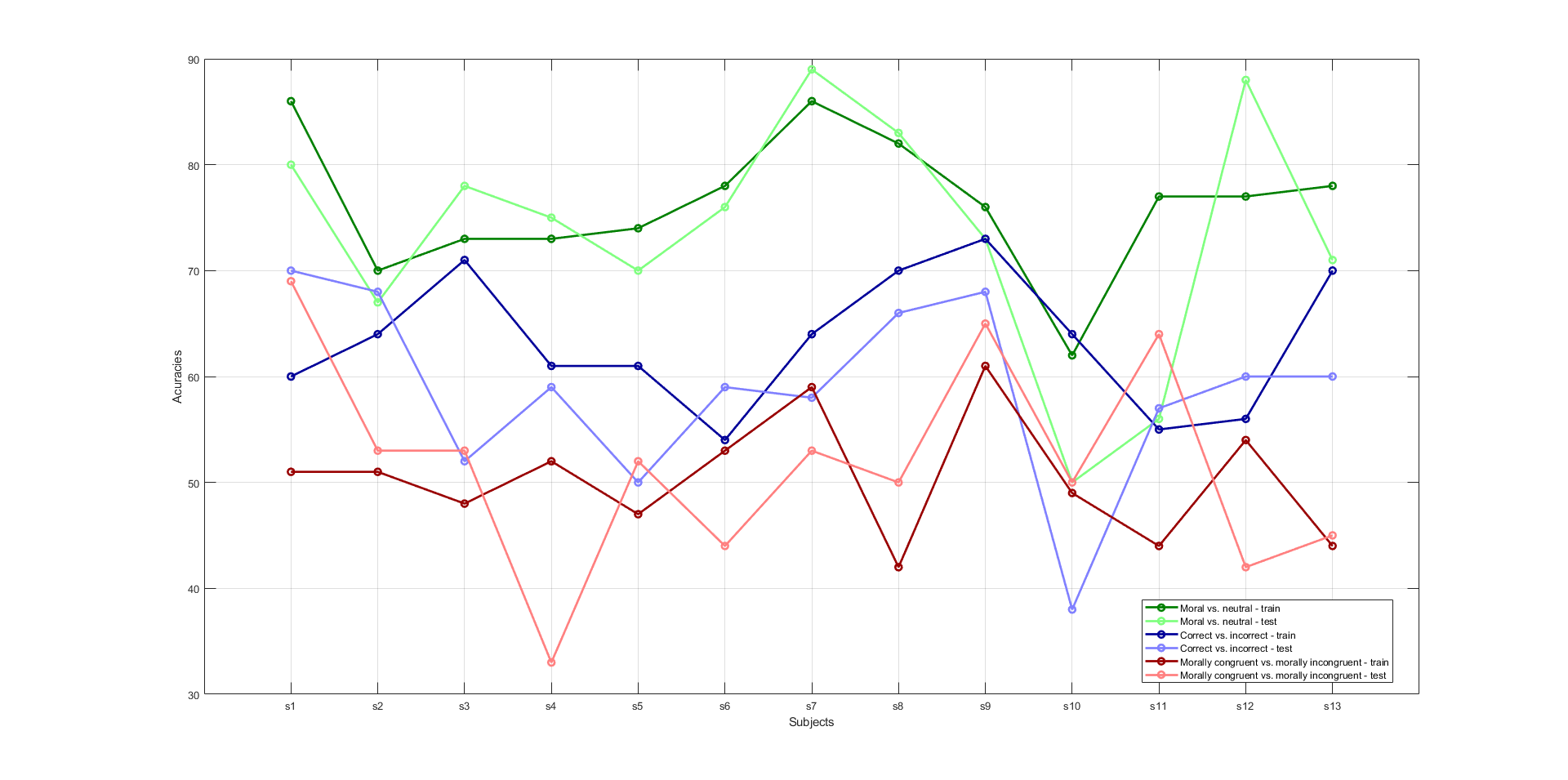


**Supplementary Figure 5.** Training and testing classification accuracies across individual subjects obtained on brain-component data for each of the three classification analyses: moral versus neutral (green), morally congruent versus morally incongruent (red), and correct versus incorrect (blue). The individual results shown for the moral versus neutral classification reflect the average accuracy per subject, computed across 10 classification runs.


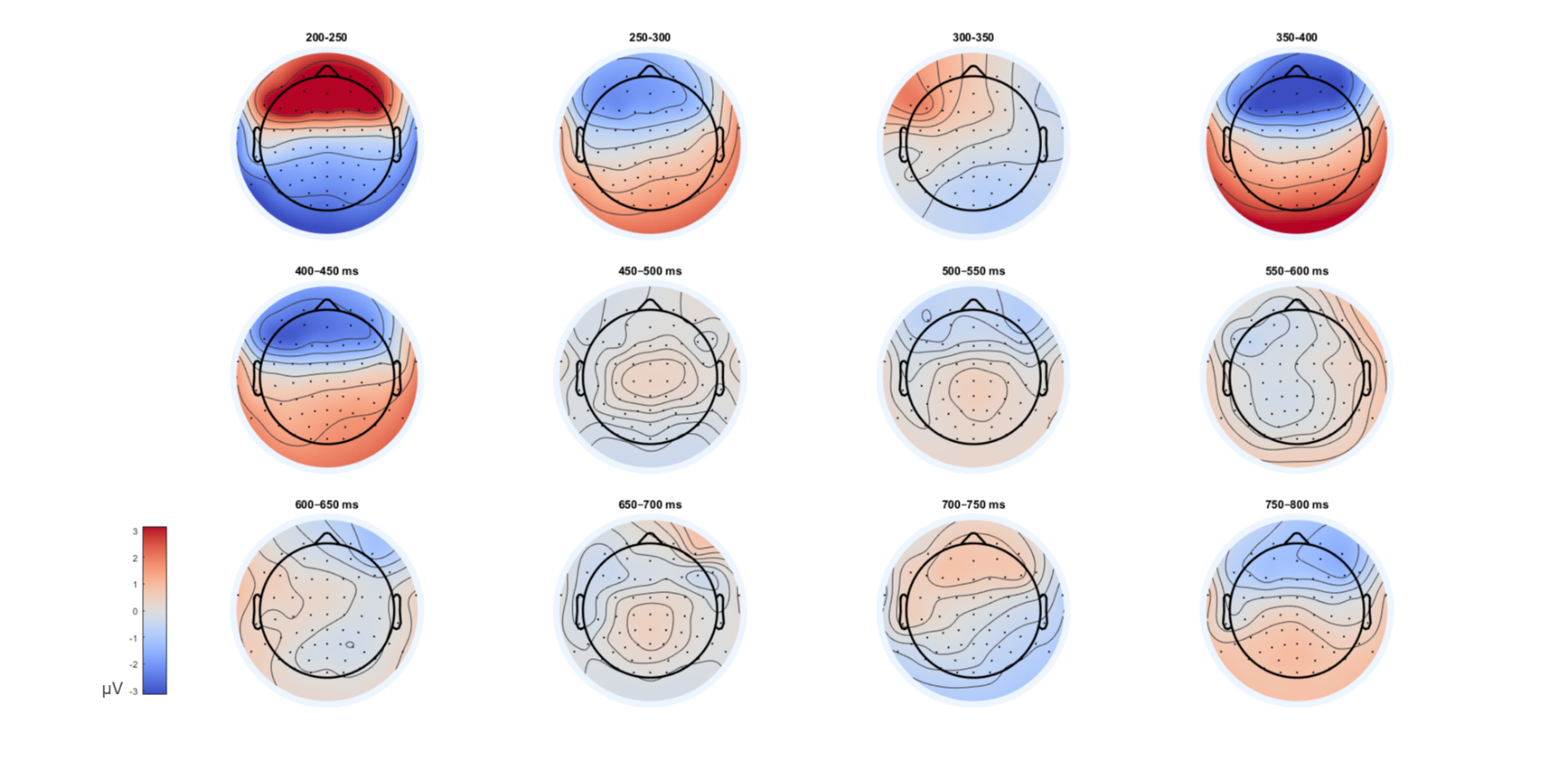


**Supplementary Figure 6.** Topographic maps illustrating activation patterns associated with the classification of moral versus neutral stimuli during training on full-component data, obtained for one of the classification runs


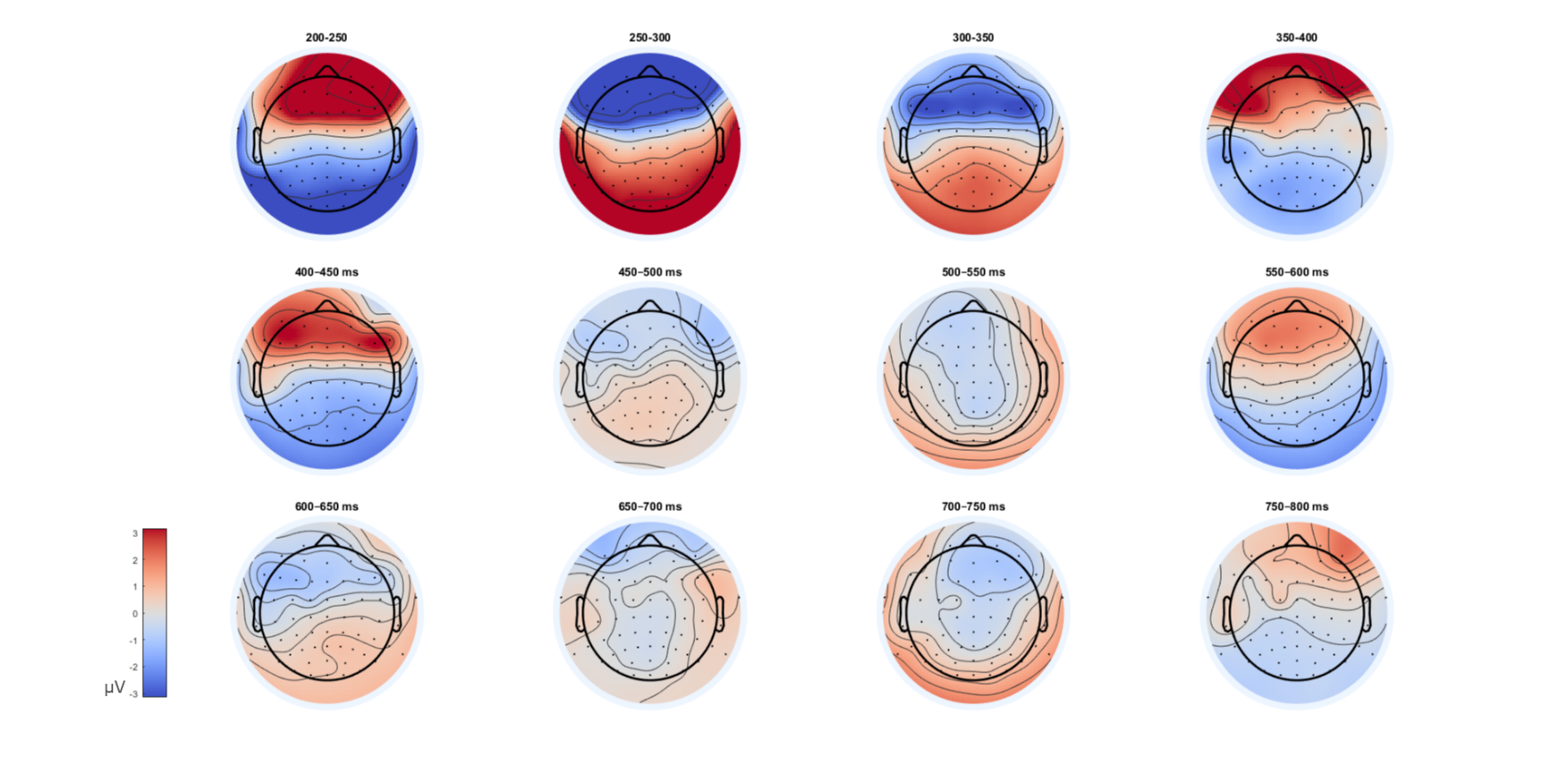


**Supplementary Figure 7.** Topographic maps illustrating activation patterns associated with the classification of morally congruent versus morally incongruent stimuli during training on full-component data

**
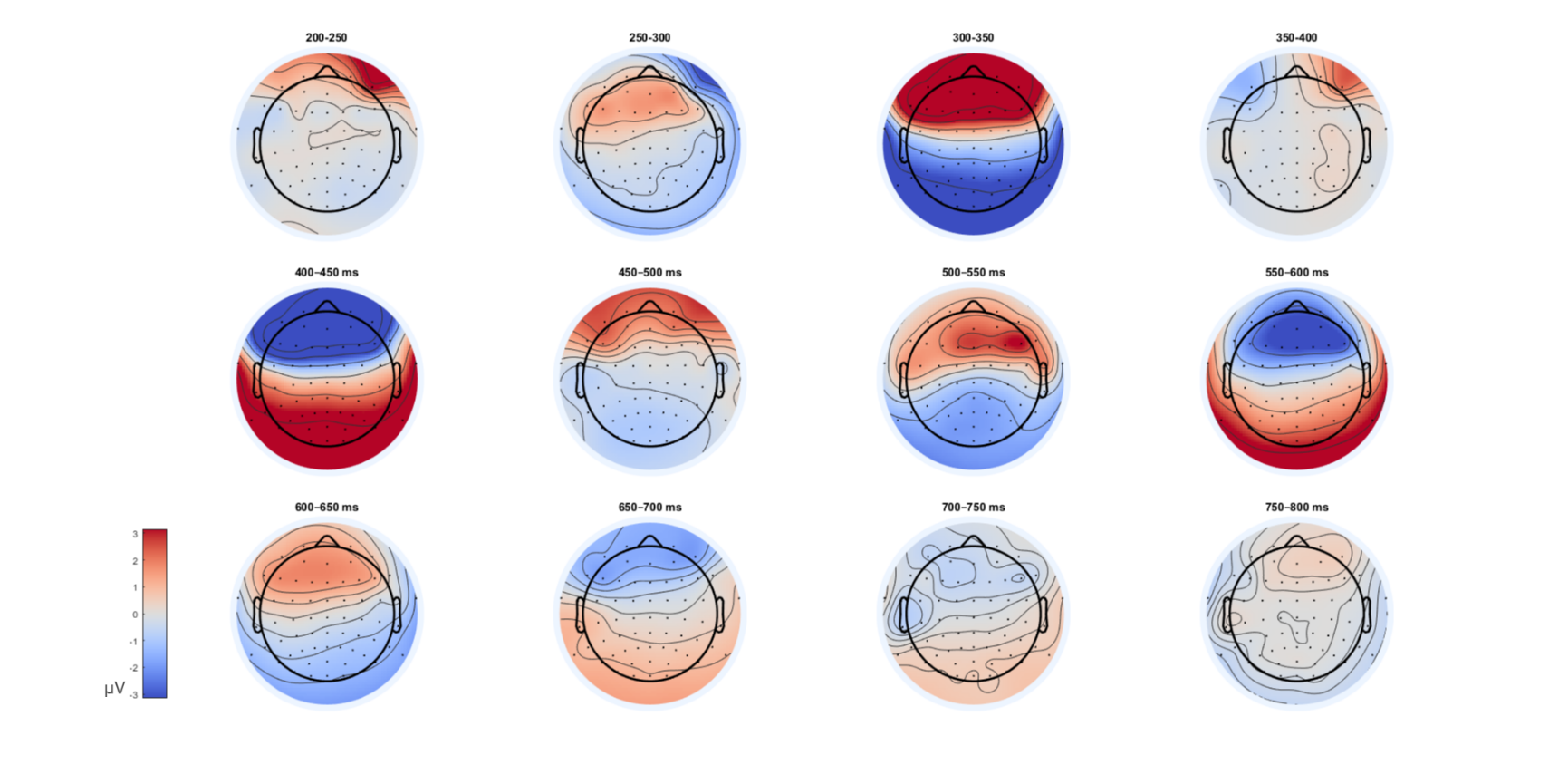
Supplementary Figure 8.** Topographic maps illustrating activation patterns associated with the classification of correct versus incorrect stimuli during training on full-component data


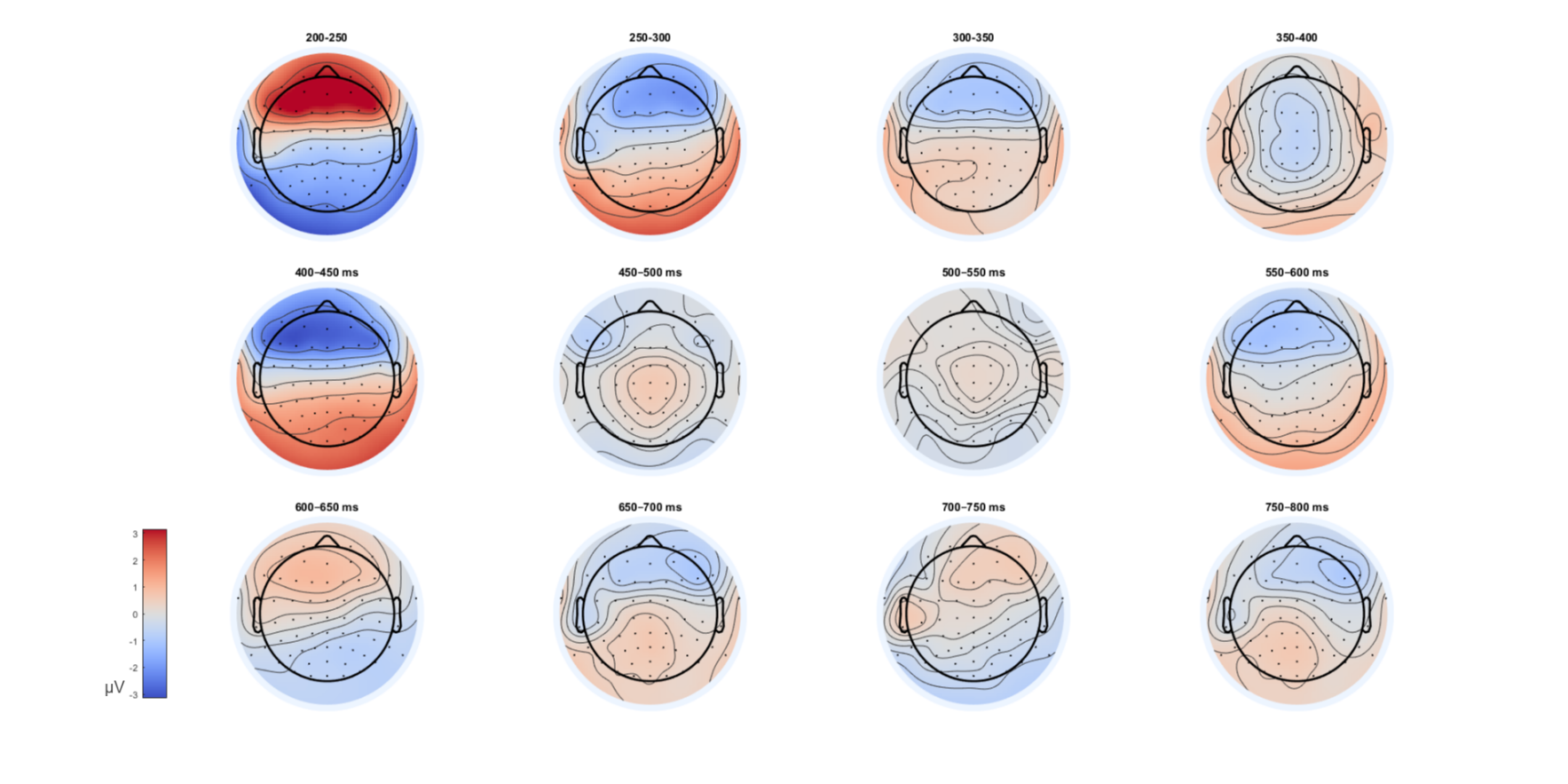


**Supplementary Figure 9.** Topographic maps illustrating activation patterns associated with the classification of moral versus neutral stimuli during training on brain-component data, obtained for one of the classification runs


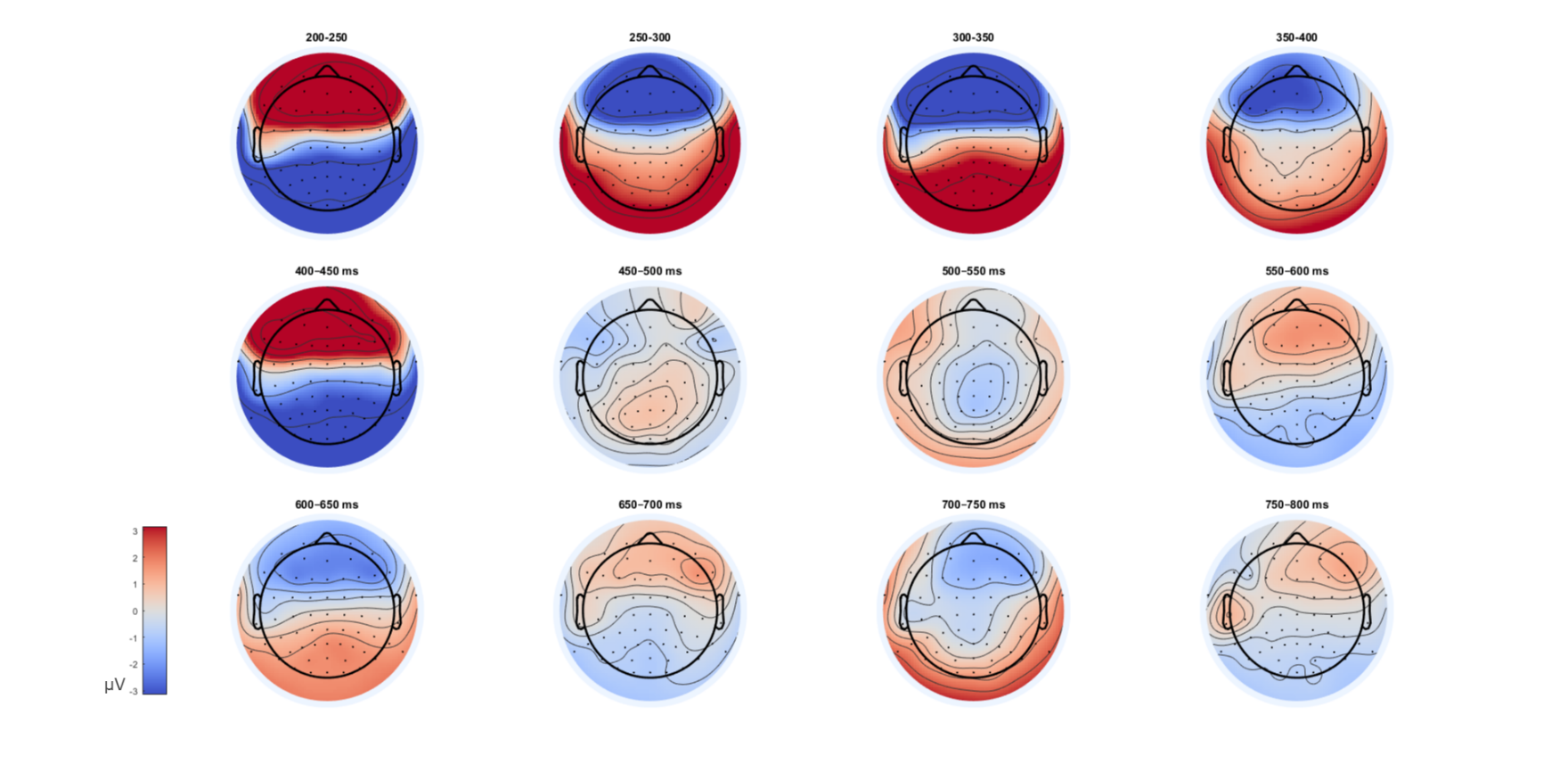


**Supplementary Figure 10.** Topographic maps illustrating activation patterns associated with the classification of morally congruent versus morally incongruent stimuli during training on brain-component data


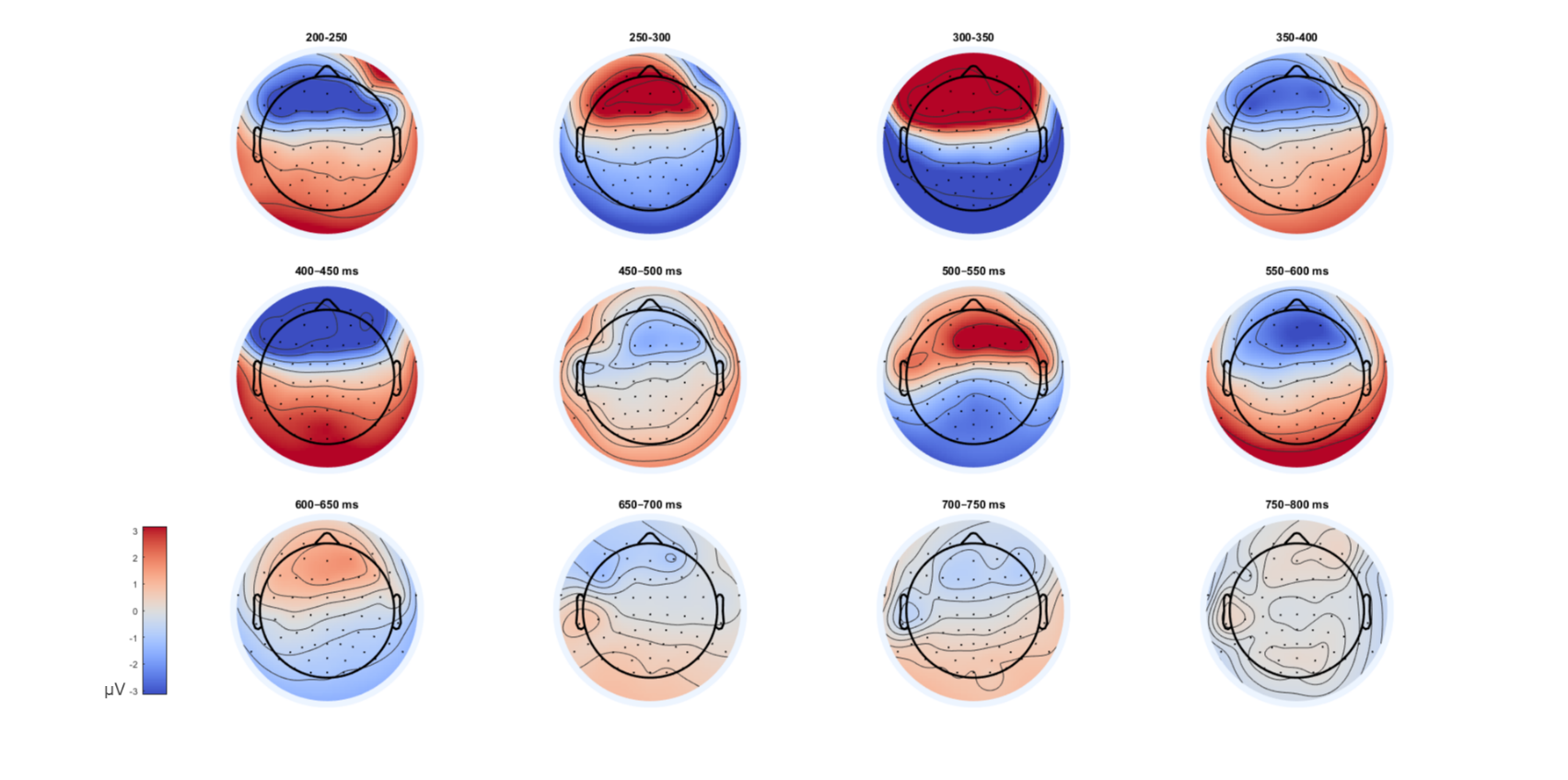


**Supplementary Figure 11.** Topographic maps illustrating activation patterns associated with the classification of correct versus incorrect stimuli during training on brain-component data
